# MR-Guided Radiotherapy: The Perfect Partner for Immunotherapy?

**DOI:** 10.3389/fonc.2020.615697

**Published:** 2021-02-02

**Authors:** Juliane Hörner-Rieber, Sebastian Klüter, Jürgen Debus, Gosse Adema, Marleen Ansems, Marcel Verheij

**Affiliations:** ^1^ Department of Radiation Oncology, Heidelberg University Hospital, Heidelberg, Germany; ^2^ Heidelberg Institute of Radiation Oncology (HIRO), Heidelberg, Germany; ^3^ National Center for Tumor Diseases (NCT), Heidelberg, Germany; ^4^ Clinical Cooperation Unit Radiation Oncology, German Cancer Research Center (DKFZ), Heidelberg, Germany; ^5^ Heidelberg Ion-Beam Therapy Center (HIT), Department of Radiation Oncology, Heidelberg University Hospital, Heidelberg, Germany; ^6^ German Cancer Consortium (DKTK), Heidelberg, Germany; ^7^ Radiotherapy & OncoImmunology Laboratory, Department of Radiation Oncology, Radboud University Medical Center, Nijmegen, Netherlands; ^8^ Department of Radiation Oncology, Radboud University Medical Center, Nijmegen, Netherlands

**Keywords:** magnetic resonance-guided radiotherapy, adaptive treatment, immunotherapy, radio-immunotherapy, preclinical

## Abstract

During the last years, preclinical and clinical studies have emerged supporting the rationale to integrate radiotherapy and immunotherapy. Radiotherapy may enhance the effects of immunotherapy by improving tumor antigen release, antigen presentation, and T-cell infiltration. Recently, magnetic resonance guided radiotherapy (MRgRT) has become clinically available. Compared to conventional radiotherapy techniques, MRgRT firstly allows for daily on-table treatment adaptation, which enables both dose escalation for increasing tumor response and superior sparing of radiosensitive organs-at-risk for reducing toxicity. The current review focuses on the potential of combining MR-guided adaptive radiotherapy with immunotherapy by providing an overview on the current status of MRgRT, latest developments in preclinical and clinical radio-immunotherapy, and the unique opportunities and challenges for MR-guided radio-immunotherapy. MRgRT might especially assist in answering open questions in radio-immunotherapy regarding optimal radiation dose, fractionation, timing of immunotherapy, appropriate irradiation volumes, and response prediction.

## Introduction

Over the last decades, substantial technical and methodological innovations in radiotherapy have enabled both more precise and focused delivery of higher doses of ionizing radiation combined with superior sparing of surrounding organs-at-risk (OARs). The latest development is magnetic resonance (MR)-guided radiotherapy (MRgRT), which bears the potential to revolutionize current standards and processes in radiotherapy. It not only offers superior soft-tissue contrast for precise detection of inter- and intrafractional changes in patient and tumor anatomy, but also allows for immediate reaction to these alterations by on-table plan adaptation (1–3). Thereby, safety margins can be reduced enabling dose escalation, while simultaneously limiting toxicity (4–7). Furthermore, some MR-linac devices offer gated dose delivery, which further facilitates irradiation of moving targets (8). Functional imaging, potentially integrated at the MR-linac, might allow for biologically guided radiotherapy to identify treatment responders, who could benefit from dose de-escalation, while additional (subvolume) boost dose might foster tumor control in non-responders (9, 10).

Despite tremendous advances in radiotherapy for improving local control and minimizing side-effects during the last decades, distant progression outside the irradiation field still remains a major challenge. Recently, immunotherapy has emerged as the fourth pillar in cancer treatment besides surgical resection, systemic therapy, and radiotherapy. Immunotherapy is increasingly regarded as a promising and attractive partner to radiotherapy, as ionizing radiation is known to inherit potent immunomodulatory effects by enhancing tumor immunogenicity and fostering immune-mediated tumor regression not only locally but also distant to the irradiation field (11, 12). However, for optimizing efficacy and reducing toxicity of anticancer radio-immunotherapy, redefinition of conventional radiotherapy volumes, doses, and fractionation schedules might be necessary (13, 14). Biologically individualized, MR-guided adaptive radio-immunotherapy might offer unique features to approach these challenges.

## Current Status of MR-Guided Radiotherapy

Hybrid systems for MRgRT, combining MR-scanners with radiotherapy devices, have first been proposed at the beginning of this century, and were introduced into clinical practice within the last years (15–17). Currently, two different systems are commercially available. Both make use of on-board magnetic resonance imaging (MRI) for patient positioning and enable treatment with step-and-shoot intensity modulated radiation therapy (IMRT) (3, 18, 19). The systems also facilitate on-table treatment plan adaption based on the actual anatomic situation at the time of treatment. As the superior soft-tissue contrast of MRI allows for precise organ-at-risk delineation and therefore enables adaptive minimization of dose to normal tissue, it is expected that MRgRT will allow dose escalation (6).

With regard to targets susceptible to breathing motion, mid-position based treatments using four-dimensional MRI acquired in treatment position directly at the MR-linac have been described (20), as well as real-time beam gating controlled by two-dimensional cine-MR (8, 21). Both strategies can contribute to a reduction of margins and thereby also potentially enable dose escalation.

First clinical data has been reported for various indications, and multiple clinical studies are ongoing that aim to show the benefits of this technology. Among others, the treated indications include liver (22, 23), pancreas (22, 24), lung (5, 25–28), prostate (4, 29–31), breast (32, 33), head and neck (16, 34), and oligometastatic disease (7, 35, 36). Although MR-guided treatments in principle can be performed in standard fractionation schemes, several authors report on the use of MRgRT for hypofractionated/stereotactic treatment schedules (4, 34, 36–38) and even single fraction regimens (39, 40).

In addition, on-board MRI at MR-linacs can also be used for quantitative MRI, thereby potentially enabling treatment response monitoring as well as treatment plan adaption based on quantitative MRI information (9).

## Current Status of Preclinical Radio-immunotherapy

Preclinical murine cancer models serve as an essential intermediate experimental model system to translate the findings from bench to bedside. In the radio-immunotherapy field, these models have extensively proven the high potential of combining radiotherapy with immunotherapy. Moreover, they have led to the identification of important underlying mechanisms. Preclinical evidence of synergy between radiotherapy and immune checkpoint blockade with anti-CTLA-4, anti-PD-1, or anti-PD-L1 has been obtained in numerous murine models of cancer (41–46). Many of the challenges of combining radiation with immunotherapy (e.g. radiotherapy dose and fractionation schedule as well as sequence of therapy) have been investigated and show that both immunogenic and non-immunogenic radiation dose and schedules exist (43, 47). It is now well-established that immunogenicity is related to sensing of cytoplasmic DNA by the cGAS/STING (cyclic-GMP-AMP synthase/stimulator of interferon genes) pathway (44, 47–49). Although these preclinical studies have provided essential new insights into the potential of radio-immunotherapy, they also have limitations. Most studies combining radiotherapy and immunotherapy only use a single ablative dose or a hypofractionated radiotherapy schedule and as a consequence the optimal timing, dose, and treatment regimen vary between models and are difficult to compare. To investigate the abscopal effect of therapy, the majority of the preclinical studies use a transplantable cell line that is injected subcutaneously in two distant locations in the mouse. In these models one tumor is irradiated and the abscopal effects are monitored in the untreated secondary tumor. In contrast to human metastatic cancer lesions, the genetic and environmental factors in the primary and secondary tumor are almost identical. These models thus may not fully recapitulate human metastatic cancer. Moreover, many small animal studies still use large field, single-beam irradiation. In these platforms, radiation exposure has limited accuracy and precision. Moreover, in-depth investigation into the anti-tumor response may be hampered by high dose radiation to healthy tissue. Data from murine experiments are important but should be carefully interpreted and used in the translation to a clinical situation. The need for more precise radiation and a growing appreciation for the role of the tumor microenvironment in anti-tumor (immune) responses has led to major developments in small animal imaging technologies (including SPECT, CT, MRI). Combining these technologies with small animal radiation research platforms enables to better mimic modern radiotherapy practice (50). Several efforts have already led to the development of small animal image-guided radiation research platforms and showed their feasibility (51–56). Both for orthotopic (55) and genetically engineered mouse models of non-small lung cancer (51) preclinical image-guided radiotherapy platforms have been set up and demonstrated their feasibility to closely mimic clinical settings. Using a xenograft model of neuroblastoma, it was shown that small animal MRI-based radiotherapy planning not only allows for precision radiotherapy, but also for accurately measuring early tumor responses which are difficult to measure by calipers (54). Orthotopic mouse pancreatic tumors were treated with image-guided radiotherapy including treatment planning techniques comparable to patient treatment (52). Additionally, for spontaneous pancreatic tumors MRI guided radiotherapy platforms have been established (53).

To achieve the best predictive value of animal-based translational cancer research, models should provide biological mechanistic insights that can be tested in a clinical setting. This requires the availability of small animal image-guided radiotherapy platforms that evolve in line with advances in the clinic and suitable models in mice with a functional immune system that mimic human responses.

## Current Status of Clinical Radio-immunotherapy

In 1953, Mole et al. were the first to describe the so-called “abscopal effect” (from the Latin prefix *ab for* “away from” and -*scopus* for “mark or target”) for the immune-mediated regression of unirradiated tumor lesions at distance from the primary site of local radiotherapy (57). However, prospective evidence for the clinical efficacy of radio-immunotherapy is still limited today (58).

Initial data is especially found in the treatment of oligometastatic cancer patients. Four phase II trials have previously demonstrated that the addition of metastasis-directed ablative therapy for all tumor sites to standard of care treatment significantly improved at least progression-free survival (PFS) or even overall survival (OS) in several different tumor entities (38, 59–62). Two recently published phase II trials included metastatic non-small cell lung cancer (NSCLC) patients treated with the anti-PD-1 antibody pembrolizumab with or without locally ablative therapies including SBRT (29, 63). The study by Theelen et al. aimed to assess whether SBRT on a single tumor site preceding pembrolizumab could enhance tumor response to immunotherapy and reported a doubled overall response for the experimental arm as compared to immunotherapy only. Although PFS was more than three times and OS more than two times higher in the SBRT arm, no significance was reached. The observation that the largest effect occurred in the PD-L1-negative subgroup suggests that radiotherapy may increase the responsiveness of non-inflamed NSCLC tumors to immune checkpoint inhibition (63). This needs further clinical evaluation. The second trial by Bauml et al. included 51 oligometastatic NSCLC patients who had received locally ablative therapy to all known sites of disease and were additionally treated with pembrolizumab. Median PFS for the locally ablative therapy arm was significantly superior with 19.1 months compared to historical controls with only 6.6 months (p = 0.005) (29).

As most current studies on MR-guided adaptive radiotherapy focus on the treatment of oligometastases, the combination of immunotherapy with MRgRT of oligometastases appears especially attractive. Henke et al. recently published results of a phase I trial of MRgRT including oligometastatic tumor lesions of different origin, while others concentrated on MRgRT of adrenal, hepatic, lymph node, or bone metastases (7, 17, 23, 35, 64, 65). Radio-immunotherapy with daily MR-guided plan adaptation bears the potential to further reduce toxicity and improve local control, while simultaneous immunotherapy might boost radiation-induced immune activation, block radiation-induced immunosuppressive effects, and eliminate microscopic disease (14).

Immunotherapy is expected to be most effective when treating patients with limited disease burden (66). Additional evidence for this hypothesis comes from the results of the PACIFIC trial, in which patients with unresectable stage III NSCLC who had responded to initial chemoradiotherapy, were treated with the anti-PD-1 antibody durvalumab (67). The addition of durvalumab nearly tripled the median PFS from 5.6 months to 17.2 months and significantly improved 2-year OS from 55.6 to 66.3% (p = 0.005). Furthermore, a *post-hoc* analysis of the KEYNOTE-001 trial demonstrated that previous radiotherapy in metastatic NSCLC patients receiving pembrolizumab significantly enhanced survival (6-months OS with radiotherapy 73% compared to 45% without) (68). Up to now, only few data are available regarding MR-guided adaptive radiotherapy for lung cancer patients (5, 25, 26). However, several studies have demonstrated a clear benefit of CT-guided adaptive radiotherapy for optimizing target coverage and sparing healthy lung tissue and hence toxicity (69–71). MR-guided adaptive radio-immunotherapy might therefore enable further dose escalation for improving local control, while simultaneously fostering the systemic immune response against distant micrometastases.

Current studies on MR-guided adaptive pulmonary radiotherapy focus on SBRT of small central and peripheral lung lesions (5, 25, 26). MR-guided adaptive SBRT of centrally or even ultracentrally located tumor lesions holds the promise to safely increase doses for such lesions adjacent to radiosensitive and vulnerable OARs (e.g. central airways, esophagus, heart). While local control following SBRT is usually satisfying, distant progression remains the major challenge (72, 73). Hence, several trials are ongoing to assess the efficacy of additional immunotherapy with SBRT for eradicating microscopic disease and fostering RT-induced immune activation in the treatment of early-stage lung cancer patients (e.g. KEYNOTE-867, PACIFIC-4). MR-guided adaptive radio-immunotherapy would further allow for safe treatment of critically located pulmonary lesions with sufficiently high dose and simultaneously reduce the occurrence of new distant tumor lesions.

As discussed above, systemic responses to immunotherapy are more frequent if overall disease burden is limited. In line with this concept, Golden et al. analyzed the occurrence of abscopal responses in metastatic patients on chemotherapy treated with concurrent radiotherapy (35 Gy in 10 fractions) to one metastatic site and granulocyte-macrophage colony-stimulating factor (12). Interestingly, the authors described that abscopal tumor responses were more frequent in patients with limited disease sites (73% in patients with only three metastases). Further support for this assertion comes from another trial, in which patients with metastatic castration-resistant prostate cancer were treated with a single dose of 8 Gy to a single bone metastasis with or without ipilimumab (74). Patients with only one osseous metastasis were more likely to benefit from immunotherapy compared to those with more bone lesions. In these scenarios, MR-guided adaptive radiotherapy could enable highly precise and focused dose delivery even to critically located tumor lesions, for which conventional techniques cannot achieve sufficiently high doses, while simultaneously potentiating local effects of immunotherapy (14).

Further tumor entities like head-and-neck tumors, rectal, cervical, or bladder cancer are expected to profit from MR-guided adaptive radiotherapy for not only enabling dose escalation, sparing of adjacent radiosensitive OARs but also for increasing the chance for organ preservation (1, 75–78). Up to now, immunotherapy is only clinically established in the treatment of metastatic tumor stages of these malignancies (79–83). Future studies are awaited to demonstrate the benefit of simultaneous radio-immunotherapy to augment local and systemic immunity and potentially reduce the risk for metastatic recurrences.

## MR-Guided Radio-immunotherapy: Challenges and Opportunities

Preclinical models suggest a window of opportunity to combine radiotherapy and immunotherapy, and early clinical studies report favorable responses to this combination. Nevertheless, many parameters remain ill-defined and need to be resolved to fully exploit the potential of radio-immunotherapy (84). These include scheduling of both modalities, fractionation regimens, treatment volume, and response prediction. The MR-linac combines unique functionalities that can address some of these outstanding questions. With regard to the optimal sequence of both modalities, preclinical data are not conclusive and suggest a combined effect that is both tumor model and immunomodulatory agent dependent. Although results from clinical studies are still scarce, the data indicate highest (local and abscopal) efficacy when radiation shortly precedes or is given during immunotherapy (67, 68, 85). Whether or not early radiation-induced influx of immune cells in the tumor microenvironment can be detected by MR imaging, e.g. as increased ADC values on DW MRI (86), and guide the optimal timing of immunotherapy, remains to be investigated.

Preclinical models imply that the dose per fraction is critical for the immunogenic effect of radiation and that a moderately hypofractionated regimen (range: 8–12 Gy per fraction) induces sufficient cytosolic double-stranded (ds)DNA to stimulate the cGAS-STING-Interferon type I pathway. Too high radiation doses (>12–18 Gy), however, can lead to the activation of feedback mechanisms, like the induction of the exonuclease Trex1 that degrades cytosolic DNA and attenuates the cGAS-STING pathway (47). This delicate biological balance between release of dsDNA and Trex1 dictates dsDNA accumulation in the cytoplasm of irradiated cells, and the subsequent initiation of anti-tumor immune responses. The dose range at which such optimal conditions arise, may turn out to be tumor specific, although in general a relatively high dose per fraction (around 8 Gy) seems required. MR-guidance is an obvious tool to safely and accurately deliver these high doses of radiation and allow the identification of the most effective fractionation regimen for synergy between radiotherapy and immunotherapy.

With respect to the ideal target volume to be irradiated, MR-based functional imaging could reveal radiosensitive or radioresistant subvolumes of tumors that may benefit from differential dosing. Intriguingly, partial tumor irradiation has been shown to elicit an effective (both local and abscopal) immune response without the need to treat the entire tumor (87, 88). High precision delivery of radiation in the context of radio-immunotherapy also involves sparing of lymphoid tissue. In fact, avoiding irradiation of tumor-associated draining lymph nodes may be crucial for the integrity of the immune response. In the context of a preclinical model comparing stereotactic radiotherapy with or without elective nodal irradiation in combination with immune checkpoint blockade, it was found that an altered T-cell chemoattractant chemokine signaling resulted in reduced immune infiltration as well as in an unfavorable balance between tumoricidal and immunosuppressive immune cells (89).

A final challenge pertains to the need for robust biomarkers of response. The superior soft tissue contrast of MR increases the ability to define the location of the tumor and adjacent normal tissues and to adapt treatment based on biological and functional dynamics of both tumors and normal structures that may occur during treatment. As responses to radio-immunotherapy will vary among tumor sites, pathological subtypes and individual patients, there is a strong clinical need for solid predictors of response to treatment. In addition to tissue-based biomarkers [such as T-cell–inflamed gene-expression profile, programmed death ligand 1 (PD-L1) expression, and tumor mutational burden], imaging-based biomarkers are emerging as promising, non-invasive, and repeatable tools that may help identify patients who have a higher likelihood of response to radio-immunotherapy across a broad spectrum of tumors. The MR-linac not only allows the use of functional MR sequences, quantitative feature extraction using radiomic approaches has become available to develop such imaging-based biomarkers, including for radio-immunotherapy. Recently, a CT-based radiomic signature was developed and validated to assess tumor-infiltrating immune cells and response to immunotherapy in patients with advanced solid tumors (90). A comparable approach using MR-based information is an obvious opportunity and will be discussed in more detail in a separate contribution to this special issue.

## Conclusions and Future Perspectives

The clinical implementation of MR-guided adaptive radiotherapy has led to new approaches to compensate for poor target definition. Superior soft tissue contrast combined with real-time plan adaptation now allows to reduce margins, increase the dose per fraction and integrate functional information in highly individualized treatment plans. These features make MR-guided radiotherapy the perfect partner for immunotherapy. Radio-immunotherapy has emerged as a promising combination for the treatment of local and abscopal disease, but the conditions for synergy need further optimization. MR-guided radiotherapy could be instrumental to address some of these variables, including optimal doses and fractionation schedules, timing of both modalities, reduced delivery volumes (partial tumor irradiation; sparing draining lymph nodes), and response prediction. This requires a collaborative effort, standardization of protocols, models, and methodologies, and a systematic collection of imaging and biomaterial data.

## Author Contributions

All authors were involved in the conception and design of this review. JH-R, SK, MA, and MV drafted the manuscript. All authors contributed to the article and approved the submitted version.

## Conflict of Interest

JH-R and SK received speaker fees and travel reimbursement from ViewRay Inc. JH-R received travel reimbursement from IntraOP Medical and Elekta Instrument AB outside the submitted work. JH-R received a grant from IntraOP Medical outside the submitted work. JD received grants from CRI—The Clinical Research Institute GmbH, View Ray Inc., Accuray International, Accuray Incorporated, RaySearch Laboratories AB, Vision RT limited, Astellas Pharma GmbH, Merck Serono GmbH, Astra Zeneca GmbH, Solution Akademie GmbH, Ergomed PLC Surrey Research Park, Siemens Healthcare GmbH, Quintiles GmbH, Pharmaceutical Research Associates GmbH, Boehringer Ingelheim Pharma GmbH Co, PTW-Freiburg Dr. Pychlau GmbH, Nanobiotix A.A., as well as IntraOP Medical outside the submitted work. JD has a research agreement with ViewRay Inc. and is member of the Scientific Advisory Board of ViewRay Inc. (Oakwood, USA). MV received grant support from Hoffmann-LaRoche, AstraZeneca, AbbVie Inc., the Dutch Colorectal Cancer Group, and the Dutch Cancer Society outside the submitted work. MV has a research agreement with Elekta Ltd.

The remaining authors declare that the research was conducted in the absence of any commercial or financial relationships that could be construed as a potential conflict of interest.
